# Cross-cultural adaptation and psychometric validation of the Indonesian version of the behavioral integrity scale for organizational settings

**DOI:** 10.1186/s40359-026-04259-1

**Published:** 2026-03-05

**Authors:** Muhammad Syarif Hidayatullah, Anissa Lestari Kadiyono, Marina Sulastiana, Yus Nugraha

**Affiliations:** Faculty of Psychology, Universitas Padjadjaran, Bandung, Jatinangor, Sumedang, West Java Indonesia

**Keywords:** Leader integrity, Trust in leadership, Perceived alignment, Psychometric adaptation, Indonesian employees

## Abstract

**Background:**

Leadership integrity is crucial in collectivist and hierarchical societies, such as Indonesia, for fostering trust and achieving organizational success. In such a high-context culture, organizational leaders’ alignment between words and actions is highly valued. However, the availability of culturally adapted measures for assessing leaders’ behavioral integrity (BI) in this context remains limited, hindering both research and practice. This study focused on the translation and cultural adaptation of the Behavioral Integrity Scale (BIS), which was initially developed by Simons et al., into Indonesian, and the assessment of its reliability and validity.

**Methods:**

Data were collected through online and offline surveys involving employees of various industry sectors in Indonesia, yielding a total of 342 responses. The validation process included evaluation of convergent and discriminant validity, confirmatory factor analysis (CFA), Content Validity Index (CVI), and Cronbach’s alpha.

**Results:**

This study’s findings indicated that the Indonesian version of the BIS retained the original structure with minor cultural adjustments. The scale demonstrates acceptable content validity (I-CVI ≥ 0.78) and (S-CVI/Ave = 0.85). CFA results showed a good fit and supported the scale’s construct validity. Reliability was excellent with Cronbach’s Alpha (α = 0.903). In addition, convergent and discriminant validity analyses strengthened the scale’s construct validity.

**Conclusion:**

The Indonesian version of BIS demonstrated good psychometric properties and appeared suitable for assessing perceived behavioral integrity of leaders in organizational settings in Indonesia. In addition to its psychometric benefits, this scale addressed the need for culturally appropriate leadership integrity measurements. This study offered practical implications for leadership development and cross-cultural research.

## Introduction

The need for integrity in human resources arises from the high level of information disclosure and the increasing critical evaluation of information. Prior research on integrity suggested that high integrity of superiors or managers is directly and positively associated with the organizational citizenship behavior of employees or subordinates [1, 2]. Conversely, low behavioral integrity of managers triggers employees to commit misconduct behaviors [1]. Behavioral integrity is significantly related to employee behavior in providing feedback to leaders and peers [3]. A meta-analysis study confirmed a strong relationship between leader behavioral integrity and employee work attitudes [4]. When leaders demonstrate behavioral integrity and managerial factors such as leadership style, skills, organizational culture, human resource management, and the ability to implement strategies effectively, employees tend to be more satisfied and committed [5].

Leader behavioral integrity can determine employee attitudes and responses in the workplace [6]. Broken promises, inconsistent messages, or hypocrisy committed by a leader can disrupt the employees’ trust in him or her. This pattern commonly unfolds within social exchange relationships [6, 7]. Decreased trust makes individuals unwilling to depend on their supervisors, lowers their emotional commitment and opinion-sharing behavior [2, 3], and triggers their cynicism, apathy, and tolerance of counterproductive behavior [8, 9]. Behavioral integrity represents a critical leadership attribute rather than merely a moral characteristic [6].

Leader behavioral integrity has significant consequences for employee attitudes and performance [6, 7]. However, most research still relies on instruments that emphasize general ethical values or moral judgments, such as ethical leadership scales or perceived leader integrity scales [10, 11]. As a result, leaders’ alignment between words and actions, especially in collectivist and non-Western environments, where moral evaluations may be shaped by cultural norms, receives less attention [12]. For this reason, this study addresses the need for a leadership integrity measurement tool that focuses on behavior and culture by adapting and validating the Behavioral Integrity Scale (BIS) in Indonesia.

Behavioral integrity measurement tools have been applied in many studies [13]. However, tools developed in Western countries may not always function effectively in different cultural environments [14]. A construct can be interpreted differently, depending on local culture, such as how people perceive leadership behavior, emotional expression, or honesty. Psychological measurements, therefore, must consider the local cultural context [15]. Collectivist values and hierarchical organizational structures in Indonesia, for example, may influence perceptions and expressions of behavioral integrity in the workplace [16, 17]. Therefore, to maintain equivalence and ecological validity of a construct, adapting measurement tools to ensure contextual appropriateness is needed [12, 18]. This study aims to adapt and validate the BIS psychometrically to ensure its applicability and interpretability in Indonesian organizational settings [13, 18]. Several researchers demonstrated that the cross-cultural adaptation of psychological instruments requires cultural, conceptual, and ecological alignment with the target context, in addition to linguistic translation [19, 20].

Various studies conducted in the last two decades mainly referred to the concept proposed by Simons [21] when discussing behavioral integrity variables. However, Simons’ concept is not the only one that has developed in understanding behavioral integrity. Table 1 presents a summary of concept patterns in interpreting behavioral integrity to guide its measurement.

**Table 1:** Concept of Behavioral Integrity (BI)

BI Core Concepts	Authors
Integrity as harmony between words and actions	Leroy et al. [22], Moorman et al. [11], Palanski & Yammarino [23], Simons [21, 24]
Integrity as ethics and morality	Bass & Steidlmeier [25], Becker [26], Craig & Gustafson [27], Mayer et al. [28]
Integrity as conformity with self (Authenticity)	Bass & Steidlmeier [25], Peterson & Seligman [29]
Integrity as a whole and reliability	Koehn [30], Palanski & Yammarino [23]

These various concepts provide diverse perspectives on behavioral integrity. Leroy et al. [22], Moorman et al. [11], Palanski and Yammarino [23], and Simons [21, 24] emphasized the harmony between words and actions. On the other hand, Bass and Steidlmeier [25], Becker [26], Craig & Gustafson [27], and Mayer et al. [28] focused more on moral and ethical aspects. Specifically, Bass and Steidlmeier [25] highlighted morality and the importance of authenticity, as suggested by Peterson and Seligman [29]. Koehn [30] and Palanski and Yammarino [23] also viewed integrity as a sense of wholeness that reflects totality, is consistent and honest in all aspects, and can survive difficult situations.

Each concept’s applicability, with their distinct advantages and disadvantages in measurement, depends on the situation and research needs. In general, there are three main approaches to measuring BI: perceptual, multidimensional, and objective approaches. The perpetual approach is represented by, among others: the Perceived Leader Integrity Scale (PLIS) [13, 27], which utilizes surveys to assess the alignment between a leader’s words and actions. The multidimensional approach adds a moral behavior dimension that evaluates the alignment of a leader’s actions with pluralistic ethical standards, including fairness, justice, and integrity [11]. The objective approach, as described by Becker, involves third-party assessment through observation or work simulation [26].

However, many existing instruments, such as the Ethical Leadership Scale (ELS) and PLIS [31], rely on subjective moral judgments, which have been shown to vary more widely in collectivist societies than in individualistic societies [32, 33]. In this context, the BIS [21], which is more observable and behavior-based, offers a reliable and culturally applicable alternative. Cross-linguistic studies utilizing artificial intelligence (AI) for moral assessment suggest that without human validation, essential details may be overlooked [34]. This highlights the necessity of behavior-based tools, such as the BIS. Additionally, recent adaptation research in Indonesia, such as the Brief Self-Assessed Wisdom Scale (BSAWS), emphasizes comprehensive psychometric validity and cultural adaptation [33]. In this context, the validation of BIS in non-Western settings in our study contributes theoretically and methodologically, enhancing the cross-cultural applicability of the behavioral integrity construct.

The approach of Simons [21, 24] is referenced and used more often due to its focus on others’ perceptions of the alignment between words and actions, which allows for more operationalized assessments and considers the external impact of one’s behavior. Meanwhile, the objective approach of Becker [26] and the pluralistic ethics approach of Moorman et al. [11] can be more challenging to measure. The challenge lies in their involvement of individual perspectives and pluralistic ethical interpretations by respondents when judging a figure, especially in the context of Indonesia, where being open to issues that people perceive as socially undesirable is challenging.

Language is crucial for ensuring conceptual, linguistic, and functional equivalence of psychological instruments during cross-cultural adaptation [18]. Accurate translation is not only vital to understanding behavioral integrity but also for preserving contextual meaning and sociolinguistic nuances [12]. Adapted measurement tools can result in semantic distortions that undermine validity and cross-cultural comparability if these factors are not carefully considered [35]. Incorporating the opinions of local experts and potential users of the instrument improves cultural relevance, ecological validity, and acceptance of the adapted scale in the organizational context where it is applied [18].

Given that behavioral integrity is defined as employees’ perceptions of leaders’ alignment between words and actions [6, 13, 21], this study focuses on respondents who have direct supervisory relationships and sufficient exposure to their leaders’ behavior. The objective is to ensure that participants can form informed judgments about leaders’ behavioral integrity based on continuous, observable interactions.

This study aims to adapt and validate the Behavioral Integrity Scale (BIS) by Simons [13] in Indonesian. The final Indonesian version of BIS will be tested for content validity using the Content Validity Index (CVI), construct validity with Confirmatory Factor Analysis (CFA), and convergent validity by analyzing the relationship between behavioral integrity and job satisfaction. According to previous research, behavioral integrity has a significant relationship with job satisfaction [36, 37]. Reliability is tested using Cronbach’s alpha.

## Methods

### Procedures

This study adheres to the guidelines of the International Test Commission (ITC) for the translation and adaptation of tests [38]. The first stage was to obtain permission from Tony Simons, the original developer of the Behavioral Integrity Scale (BIS), to adapt the BIS for use in Indonesia. Before adaptation, a literature review was conducted to assess the measurement tool related to the integrity of leader behavior. The scale chosen was conceptually compatible with the research objectives, and no Indonesian version was available.

The adaptation process involved forward translation and back translation by translators selected based on their educational background, language proficiency, cultural understanding, knowledge of psychometrics, and experience in the field of psychology. Expert reviewers were invited to evaluate the equivalence between the concepts and meanings in the adapted and original items. A readability test and a field test were also conducted to assess comprehensibility.

Data were collected through online and offline surveys, using Google Forms (Google LLC, Mountain View, CA, USA) for online responses and paper and pen for offline collection. An online scale distribution was conducted through social media platforms, including WhatsApp and Instagram, targeting employees working in companies in Indonesia. The snowball sampling method was also used for offline scale distribution, where acquaintances who met the criteria were invited to participate and encouraged to share the scale with their colleagues.

Participation in this study was voluntary. All potential participants were informed about the study objectives and provided informed consent prior to participation. Not everyone contacted gave agreement to participate in the study. Those who refused or did not complete the questionnaire were excluded from the final dataset without consequence. Participants who completed the survey received a gift of IDR 10,000 as a token of appreciation, which can be redeemed as a mobile phone or e-wallet balance. The incentive served as compensation for participants’ time and was not contingent on their answers.

### Participants

Participants in this study were recruited through convenience sampling. They were employees of Indonesian organizations who had a direct supervisory relationship and had worked under their leaders for at least six months. This condition was set to ensure their sufficient exposure to leadership behavior. Individuals without a direct supervisor were not eligible to participate.

The participants in this study consisted of 342 employees from private, regional, or state-owned companies (excluding civil servants and government employees). A study examining a measurement tool factorial structure requires a large sample size of approximately 300 respondents or more [39]. A sample should be ≥ 200, with a ratio of at least 5:1, ideally 10:1, or up to 20:1 per variable. A smaller sample is acceptable for initial exploration, such as questionnaire trials [40]. In this study, eight items were to be analyzed to meet the minimum number of participants recommended.

Based on the demographic data obtained in this study, most participants were female, comprising 71.3% (*N* = 244) of the sample, whereas males represented 28.7% (*N* = 98). The majority of participants were aged 25–34 years, constituting 60.5% (*N* = 207). Furthermore, participants aged 18–24 years represented 24.3% (*N* = 83), those aged 35–44 years 13.5% (*N* = 46), and those aged 45–54 years 1.8% (*N* = 6). Based on the length of service, 5.9% (*N* = 20) had worked for less than one year, 15.2% (*N* = 52) for 1–3 years, and 12.6% (*N* = 43) for 3–5 years. Furthermore, participants with 5–10 years of work experience constituted 24.8% (*N* = 85), indicating that most participants had had sufficient work experience. Meanwhile, 21.1% (*N* = 72) of the participants had 10–15 years of work experience. The remaining 20.4% (*N* = 70) had worked for over 15 years. Meanwhile, in terms of position, the majority were staff or operational-level employees, namely 91.5% (*N* = 313). Meanwhile, 6.7% (*N* = 23) held managerial roles. Another small portion, 1.8% (*N* = 6), was in technical professions. The most significant proportion of respondents came from the manufacturing sector (22.2%, *N* = 76), hospitality and food services (14.6%, *N* = 50), agriculture, forestry, and fisheries (9.1%, *N* = 31), financial and insurance services (8.5%, *N* = 29), wholesale and retail trade (8.2%, *N* = 28), information and communication (5.6%, *N* = 19), transportation and warehousing (5.3%, *N* = 18), construction (3.5%, *N* = 12), and mining and quarrying (1.8%, *N* = 6). The remaining 21.3% (*N* = 73) were spread across various fields, including logistics, education, utilities, property, healthcare, personal services, and small-scale service and trade businesses. This distribution reflected the heterogeneity of organizational contexts.

### Study measures

The Behavioral Integrity Scale (BIS), developed by Simons et al. [13], measures leaders’ behavioral integrity and has been consistently conceptualized as a unidimensional construct [13] in previous research. The Behavioral Integrity Measurement Scale uses a 5-point Likert scale, ranging from 5 (strongly agree) to 1 (strongly disagree). Behavioral integrity is measured using an eight-item scale. The full Indonesian version of the BIS is provided in Appendix A.

The job satisfaction (JS) measurement tool, developed by Cooper et al. [40]. and adapted by Hapsari [41] into Indonesian, was used in this study. The reported reliability test results showed that the intrinsic job satisfaction factor had a coefficient of *α* = 0.81, while the extrinsic job satisfaction factor had a coefficient of *α* = 0.73. This measurement tool consisted of 10 items, including five that assessed intrinsic aspects, four that assessed extrinsic aspects, and one that evaluated overall job satisfaction. The responses to the items in this measurement tool were as follows: 0 = very dissatisfied, 1 = somewhat dissatisfied, 2 = not sure, 3 = somewhat satisfied, 4 = very satisfied.

### Data analysis

Descriptive statistics were used to summarize the participants’ socio-demographic characteristics, including gender, age, education level, length of service, industry sector, and job position. Frequency and percentage were used for categorical variables, while Mean (*M*) and Standard Deviation (*SD*) were used to summarize continuous variables. This approach is standard for describing sample composition and assessing sample heterogeneity prior to psychometric procedures and factor analysis [40, 42].

Content validity was evaluated using the Content Validity Index (CVI), a widely recommended method for assessing the relevance and clarity of cross-cultural adaptations of psychological instruments [43–45], by performing two types of calculations: I-CVI (Item Content Validity Index) and S-CVI (Scale Content Validity Index). The content validity assessment was carried out by five experts who evaluated the original scale and its translation. The five experts were deliberately selected based on their educational background in psychology and experience in psychometrics, psychological measurement, and cross-cultural test adaptation [18, 43, 44]. They were invited through direct communication with each of them. They also had previous experience in evaluating the content validity of psychological instruments in Indonesia. In addition, they were all lecturers. The experts were asked to rate on a 4-point scale of relevance (1 = not relevant, 2 = somewhat relevant, 3 = quite relevant, and 4 = very relevant) and clarity (1 = unclear, 2 = item needs revision, 3 = clear, but needs minor revision, and 4 = very clear). The I-CVI threshold of 0.78 was applied because it is recommended when three or more experts are involved, while S-CVI/Ave values of 0.80 or higher indicate acceptable content validity and values of 0.90 or higher indicate excellent content validity [43–45].

Confirmatory Factor Analysis (CFA) was used instead of Exploratory Factor Analysis (EFA) because the Behavioral Integrity Scale (BIS) has a theoretically and empirically established unidimensional structure. CFA is recommended when the factor structure has been predetermined, and the main objective is to examine construct validity in a new cultural context [40, 42, 46]. Because chi-square statistics are highly sensitive to sample size, model fit was evaluated using several complementary fit indices, in accordance with recommendations in the structural equation modeling literature [40, 42, 47].

Convergent and discriminant validity were evaluated using Average Variance Extracted (AVE) and the Fornell-Larcker criteria, as these methods are widely recommended for assessing construct validity in CFA-based measurement models [40, 42, 48]. Convergent validity was also evaluated using the relationship between BIS and job satisfaction. Reliability was evaluated using Cronbach’s alpha, the most commonly used index of internal consistency in studies of adaptation and validation of psychological scales [40, 42].

### Ethical consideration

The Universitas Padjadjaran Research Ethics Committee Permission Letter No. 376/UN6.KEP/EC/2025 and informed consent ensured the ethical conduct of this study.

## Results

### Demographic

The results of the multiple linear regression analysis and ANOVA indicated that demographic variables had a minimal influence on the perceived leader behavior integrity (PLBI). The regression model explained only 3.6% of the variance (*R*² = 0.036, adjusted *R*² = 0.024), indicating that demographic factors were a weak predictor of PLBI. Descriptive statistics indicated that male employees in managerial positions had a slightly lower PLBI score (*M* = 30.14, *SD* = 7.037) compared to female employees (*M* = 36.00, *SD* = 4.213). Staff-level employees showed a lower PLBI score (*M* = 31.22, *SD* = 5.347) than individuals with specific job roles (*M* = 36.17, *SD* = 3.601). However, this difference did not reach statistical significance. The ANOVA results indicated that position alone was insufficient to predict PLBI; a significant interaction was found between gender and position (*p* = 0.008), where women in managerial positions had PLBI scores (*M* = 36.00, *SD* = 4.213) higher than men’s (*M* = 30.14, *SD* = 7.037). These data indicate that gender moderates the relationship between position and PLBI. Position in this study was the only significant demographic predictor for PLBI (*B* = 1.742, *p* = 0.038). Still, it was moderated by gender, with managerial employees having a higher perception of leader integrity than staff. At the same time, job specifics were not tested separately due to the small sample size. Table 2 presents descriptive statistics, and Table 3 presents the ANOVA and regression analysis for PLBI.

**Table 2:** Descriptive statistics and ANOVA

Variables	*N*	Mean PLBI	SD	ANOVA F-value	*p*-value	Partial η^2^
Total	342	31.39	5.45	-	-	-
Gender: Male	98	32.21	5.4	0.069	0.793	0.000
Gender: Female	244	31.06	5.44	0.069	0.793	0.000
Job: Staff	313	31.22	5.35	2.794	0.063	0.016
Job: Managerial	23	32.43	6.65	2.794	0.063	0.016
Job: Specific	6	36.17	3.6	2.794	0.063	0.016
Gender: Male (Staff)	81	32.37	5.0	2.794	0.063	0.016
Gender: Female (Staff)	232	30.82	5.41	2.794	0.063	0.016
Gender: Male (Managerial)	14	30.14	7.04	4.925	0.008	0.028
Gender: Female (Managerial)	9	36.0	4.21	4.925	0.008	0.028
Gender: Male (Spesific)	3	37.67	4.04	-	-	-
Gender: Female (Specific)	3	34.67	3.05	-	-	-

Note: *PLBI *Perceived Leader Behavioral Integrity

**Table 3:** Regression analysis

Predictor	B	SE B	β	t	*p*	95% CI Lower	95% CI Upper
(Constant)	34.9	2.386	-	14.628**	<0.001	30.207	39.594
Gender	-1.231	0.669	-0.102	-1.841	0.066	-2.546	0.084
Age	-0.112	0.059	-0.118	-1.917	0.056	-0.227	0.003
Tenure	-0.002	0.009	-0.015	-0.241	0.810	-0.019	0.015
Job	1.742	0.838	0.114	2.078*	0.038	0.093	3.39
Gender x Job	1.56	0.543	0.093	2.876**	0.008	0.493	2.627
Model Fit	*R*^2^ = 0.036 Adjusted *R*^2^ = 0.024						

*p<0.05, **p<0.01

### Content validity

This study used the Content Validity Index (CVI) method to calculate the validity of the content. Two types of calculations were used to measure validity: I-CVI (Item-Level Content Validity Index) and S-CVI/Ave (Scale-Level Content Validity Index, Averaging Method). The calculation results for all items had an I-CVI ≥ of 0.78, with an S-CVI/Ave of 0.85, making it valid with a minimum limit of 0.80 [45]. Additionally, an assessment of the clarity aspect was conducted using the same method [49], yielding a score of 0.825. Nevertheless, revisions based on input from expert reviewers, including replacing the term “manajer” with “atasan” to adjust the scope of the position and using more general terms, such as replacing “kesinambungan” with “kesesuaian,” were made.

### Reliability and validity of the Behavioral Integrity Scale (BIS)

The reliability and validity of the BIS were evaluated using Cronbach’s Alpha, item analysis, and Confirmatory Factor Analysis. The internal consistency reliability test yielded a Cronbach’s Alpha of 0.903, exceeding the recommended threshold for psychological scales [40, 42, 46]. Item analysis was performed using corrected item-total correlation (CRIT) and factor loadings analysis. All items met the recommended thresholds of CRIT ≥ 0.30 and factor loading ≥ 0.40, confirming good item discrimination and construct validity [40, 42, 46]. The detailed item analysis results are shown in Table 4.

**Table 4:** Item analysis

Item	CRIT	Factor Loadings
Item 1	0.707	0.660
Item 2	0.684	0.611
Item 3	0.677	0.610
Item 4	0.635	0.637
Item 5	0.739	0.676
Item 6	0.696	0.632
Item 7	0.719	0.713
Item 8	0.695	0.627

Note: *CRIT* Corrected item-total correlation. Factor loadings were obtained from Confirmatory Factor Analysis (CFA). Items with CRIT ≥ 0.30 for good item discrimination and factor loadings ≥ 0.40 for acceptable construct validity [40, 42, 46]

CFA was conducted using a single-factor model, in line with previous empirical research [13]. EFA was not undertaken because BIS has been considered a one-dimensional construct both theoretically and practically. This study aimed to confirm this structure in various cultural contexts. The initial CFA results showed acceptable fit on several indices (GFI = 0.991, SRMR = 0.04, IFI = 0.927, NFI = 0.915, CFI = 0.927) but showed poor fit on RMSEA = 0.124 and significant chi-square statistics. Residual covariances between items 1 and 2, 7 and 8, and 6 and 8 were added based on modification indices. Such model respecifications were applied only when theoretically justified and reflecting overlap in item content or formulation, a practice discussed in confirmatory factor analysis, particularly in cross-cultural validation [42, 50]. After model adjustment, the RMSEA improved to 0.078, and the other indices (GFI = 0.996, SRMR = 0.027, IFI = 0.976, NFI = 0.964, CFI = 0.976) also showed improved values. This modified model showed excellent fit across various parameters (see Table 5). All factor loadings in the modified model remained above the recommended threshold of ≥ 0.40, confirming item adequacy [40]. In addition, the internal consistency of the construct was reinforced by composite reliability (CR = 0.847), which exceeded the recommended threshold of 0.70 [40].

**Table 5:** Initial and modified CFA model fit indices

Fit Parameter	Initial	Modified	Criterion
Chi square (*X*^2^, df), *p*-Value	123.673 (20), < 0.001	52.099 (17), < 0.001	≥ 0.05
Goodness of fit index (GFI)	0.991	0.996	≥ 0.90
Root mean square error of approximation (RMSEA)	0.124	0.078	< 0.08
Standardized root mean square residual (SRMR)	0.04	0.027	< 0.08
Normed fit index (NFI)	0.915	0.964	≥ 0.90
Incremental fit Index (IFI)	0.927	0.976	≥ 0.90
Comparative fit index (CFI)	0.927	0.976	≥ 0.90

Note: Model fit indices were evaluated based on commonly recommended thresholds [40, 47, 51, 52]. To improve model fit, residual covariances were added between items 1 and 2, 7 and 8, and 6 and 8, based on the modification indices

### Convergent and discriminant validity

The convergent validity of the BIS was assessed in two ways. First, the scale showed a positive and significant correlation with job satisfaction (*r* = 0.635), in line with theoretical expectations (see Table 6). Second, the Average Variance Extracted (AVE) values for the BIS exceeded the recommended threshold of 0.50, further supporting convergent validity [40, 48]. Discriminant validity was evaluated using the Fornell-Larcker criteria (see Table 7). This was met because the AVE values for BI (0.527) and JS (0.482) were greater than their square correlations (*r*^²^ = 0.403), indicating that these constructs are empirically distinct [40, 42, 48].

**Table 6:** Correlation between behavioral integrity and job satisfaction

	PLBI	JS
PLBI	1	
JS	0.635**	1

Note: **p < 0.01

**Table 7:** AVE and discriminant validity of constructs

Construct	AVE	*R* (BI-JS)	*r* ^2^
PLBI	0.527	0.635	0.403
JS	0.482	0.635	0.403

Note: Convergent validity is supported when AVE ≥ 0.50, and discriminant validity is satisfied if AVE > *r*^2^ [40, 42, 48]

## Discussion

This study aimed to translate, culturally adapt, and psychometrically validate the Behavioral Integrity Scale (BIS) for use in Indonesian organizational settings. The findings demonstrated that the Indonesian version of the BIS exhibits good psychometric properties, including excellent internal consistency, satisfactory content validity, and adequate construct validity. The unidimensional structure of the BIS was supported by confirmatory factor analysis, and model fit was acceptable after theoretically justified model refinements. In addition, expert evaluation indicated that the items were relevant and clearly formulated, supporting the content validity of the adapted scale [53]. Overall, these findings indicated that the Indonesian version of the BIS was a reliable and valid instrument for assessing employees’ perceptions of leaders’ behavioral integrity.

The adaptation process also demonstrated the importance of cultural and linguistic sensitivity for scale validation in Indonesia. Specifically, expert reviewers suggested replacing the term “manajer” with “atasan” to better reflect the broader and more commonly understood notion of leadership in Indonesian organizations. This adjustment improved the clarity, inclusiveness, and ecological validity of the scale, consistent with findings from previous Indonesian validation studies emphasizing culturally appropriate terminology [54].

The present findings further supported the cross-cultural and ecological validity of the BIS, in line with evidence from other collectivist cultures such as South Korea and China [7, 9, 21, 55–57]. Prior studies have consistently demonstrated that leader behavioral integrity is positively associated with important organizational outcomes, including job performance, organizational citizenship behavior, and proactive work behavior [7, 57, 58]. Together, these findings emphasized the importance of behavioral consistency as a leadership attribute across various cultural contexts, both theoretically and practically.

In addition to its contribution to psychometrics, this study highlights the importance of assessing leadership integrity through observable behavior alignment rather than abstract moral judgment. Unlike previous instruments such as PLIS [27] and ELS [31], which emphasize subjective moral judgments and broader constructions of ethical leadership, the BIS focuses on the consistency between leaders’ words and actions. This distinction is particularly important in collectivist and highly contextual societies such as Indonesia, where communication relies heavily on implicit cues, shared norms, and relational harmony rather than direct verbal expression [59, 60]. In such contexts, individuals in subordinate positions tend not to express disagreement verbally and prefer to assess integrity through observable behavior. Discussions on cross-cultural measurement [12, 19, 20] emphasize that instruments prioritizing observable behavior tend to exhibit ecological validity and are more culturally transferable, further reinforcing the appropriateness of the BIS in Indonesian organizational settings.

Theoretically, this study contributes to the literature by confirming that behavioral integrity can be understood as a construct that is observable across cultures. Moral integrity differs from other constructs in that it is often culture-bound and susceptible to varying normative judgments [27, 31]. In contrast, behavioral integrity operationalized through BIS appears to be more clearly and broadly interpreted in various contexts [13, 21]. This study provides evidence supporting the validity of BIS in the Indonesian context, reinforcing the argument that behavior-based constructs offer higher cross-cultural transferability, consistent with cross-cultural organizational behavior theory [12, 61]. This study presents a psychometrically valid Indonesian version of the BIS, thereby contributing to the discussion on the ecological validity of behavioral integrity across contexts.

The model fit index showed that most indicators met acceptable criteria. However, the RMSEA value indicated the need for model refinement as a general and essential step in cross-cultural adaptation to psychological measurement tools [18, 62]. Modification indices recommended adding residual covariance between several item pairs (items 1 and 2, 7 and 8, 6 and 8). After applying this adjustment, the RMSEA decreased to 0.078, indicating a better fit between the theoretical construct and the observed data characteristics [42]. In the Indonesian context, this modification reflects how employees interpret leaders’ behavior through relational and contextual cues, rather than through isolated statements. The pattern is consistent with high-context communication, where meaning depends on shared norms and implicit understanding rather than explicit words [60, 63, 64]. The correlated residuals between items 1–2, 7–8, and 6–8 indicated that respondents considered these items to be semantically overlapping due to similarities in expression and structure, so that these items might be regarded as functionally in a high-context setting, which explained the initial mismatch and showed that the refinement of the model reflect both statistical adjustments and cultural-linguistic interpretations of semantic scope [65–67]. The contemporary cultural framework suggested that in high-context societies, the formation of relational meaning reinforced the importance of observable consistency between leaders’ words and actions in assessing their integrity [59, 68, 69].

Reliability analysis indicated that the Indonesian version of the Behavioral Integrity Scale (BIS) exhibited excellent internal consistency. The results of this study aligned with those of previous studies, which suggested that this scale retained its psychometric strength across diverse cultural environments [6, 11, 13, 21].

The findings of this study were consistent with previous studies that found the role of leader integrity in fostering positive attitudes. The perception of leader behavioral integrity, as measured using the Behavioral Integrity Scale (BIS), showed a significant positive correlation with job satisfaction [8, 70, 71], providing evidence of the convergent validity of the Indonesian version of the BIS scale. Furthermore, the results satisfied the Fornell-Larcker criterion, thereby affirming the scale’s discriminant validity [40, 42, 48].

This study demonstrates that BIS is relevant in the Indonesian work environment, as evidenced by high factor loadings and significant correlations with other factors. The results of this study were consistent with research findings from countries with collectivist cultures, such as China and Turkey [72–74]. Leaders who demonstrate integrity and ethical behavior will be more valued because they create a sense of security, trust, and group harmony [75]. A study by Erkutlu and Chafra [55] found that perceptions of leaders’ behavioral integrity are positively correlated with positive work attitudes.

The validated Indonesian version of the BIS can help organizations in Indonesia analyze the perceptions of leaders’ behavioral integrity. Perceptions of leadership integrity are crucial in collectivist cultures, such as those found in Indonesia [17]. Leaders who are consistent and transparent in their daily work can increase employee motivation and organizational effectiveness [10, 75].

In practice, this scale can be applied in leadership development programs, performance appraisals, and organizational surveys to identify strengths and weaknesses in the relationship between leaders and employees [76, 77]. In hierarchical and collectivist cultures, where direct criticism of leaders is often avoided, the BIS provides employees with a structured way to express their perceptions of integrity without causing confrontation [17, 78]. The implementation of this tool may help organizations foster trust, support work engagement, and potentially enhance organizational effectiveness [21, 79].

### Study limitations

The Indonesian version of the BIS study has produced sufficient support for validity and reliability. Still, respondent proportionality needs to be further secured to ensure generalization, especially regarding gender and position. The external validity and generalizability of a measurement tool are essential to test in various populations and environmental contexts [46]. Sampling strategies that rely on convenience and snowball techniques also have the potential to limit the representation of samples across various industries [80, 81]. Although participants came from a variety of fields, some industries were underrepresented, which may affect the generalizability of the findings. Future studies should consider a more equitable distribution of samples across occupational fields.

In addition, the predictive validity of the Indonesian version of the BIS was not evaluated in this study. However, content validity, construct validity, convergent validity, and internal consistency reliability have been established. This limitation is understandable given that the primary focus of this study was psychometric validation and cross-cultural adaptation. Further research is recommended to evaluate the predictive validity of the BIS by examining its ability to predict relevant work outcomes such as job performance, organizational citizenship behavior, work engagement, or counterproductive work behavior, in line with the recommended scale validation stages following cross-cultural adaptation [19, 46]. With such efforts, the scale will be further strengthened in its practical usefulness and relevance to organizational research and practice.

## Conclusions

The Behavioral Integrity Scale (BIS) was translated and culturally adapted into Indonesian in accordance with the International Test Commission (ITC) guidelines. The study’s validity test results demonstrated the good psychometric properties of the Indonesian version of the BIS, with good internal consistency and adequate corrected item-total correlation (CRIT) and factor loadings.

These results suggested that the Indonesian version of the BIS is a valid and reliable instrument for assessing perceptions of a leader’s behavioral integrity in organizational settings in Indonesia. This scale is also helpful for further research and the diagnosis of leadership effectiveness. It can be compared with research results from other countries. Future research is recommended to validate this scale in various sectors, roles, and areas. 

### Ethical approval

Before participating in this study, each respondent provided their informed consent, which included the option to publish their response anonymously. All procedures were conducted in accordance with the ethical standards outlined in the 1964 Declaration of Helsinki and its subsequent amendments, or with equivalent ethical standards. Ethical approval from the ethics committee at Universitas Padjadjaran, Bandung, Indonesia, ensured that all research procedures were carried out in accordance with established norms.

Data were collected offline and online. Researchers and enumerators conducted offline data collection in person. WhatsApp and Instagram were only used to disseminate links to the online recruitment questionnaire. No user-generated materials, profile data, or media posts were accessed, collected, or analyzed. The utilization of WhatsApp and Instagram adhered to their terms of service, which do not prohibit users from disseminating survey URLs for voluntary participation. Consequently, no further approvals were necessary from the platform providers.

## Data Availability

The datasets generated and analyzed during the current study are available from the corresponding author on reasonable request.

## Appendix

### Indonesian version of Behavioral Integrity Scale (BIS)

#### Instruksi

Pada skala ini, Bapak/Ibu akan diberikan 8 buah pernyataan. Silakan baca pernyataan-pernyataan di bawah ini dengan seksama, kemudian berikan respon terhadap pernyataan tersebut yang paling sesuai sebagaimana yang dihadapi atau dialami Bapak/Ibu saat ini. Berikut adalah skala penilaian yang dapat Bapak/Ibu pilih:

- (STS) Sangat Tidak Setuju
- (TS) Tidak Setuju
- (N) Netral
- (S) Setuju
- (SS) Sangat Setuju

**Table:** Tidak ada jawaban benar atau salah sehingga Bapak/Ibu dimohon untuk menjawab dengan seterbuka mungkin

No	Pernyataan	STS	TS	*N*	S	SS
1	Ada kesesuaian antara perkataan yang diucapkan dan tindakan yang dilakukan atasan saya					
2	Atasan saya menepati janjinya					
3	Atasan saya menerapkan prinsip apa yang dia jalani dalam perilakunya sehari-hari					
4	Atasan saya melakukan apa yang dia katakan sehingga tidak berupa instruksi saja tanpa contoh perilaku					
5	Atasan saya berperilaku sesuai dengan nilai-nilai yang dia ajarkan					
6	Atasan saya menunjukkan prioritas yang sama dengan yang dia jelaskan					
7	Ketika atasan saya menjanjikan sesuatu, saya bisa meyakini bahwa hal itu akan terjadi					
8	Ketika atasan saya berkata dia akan melakukan sesuatu, maka dia benar-benar akan melakukannya.					

## Abbreviations

AVE: Average Variance Extracted
BI: Behavioral Integrity
BIS: Behavioral Integrity Scale
CFA: Confirmatory Factor Analysis
CR: Composite Reliability
CRIT: Corrected Item-Total Correlation
CVI: Content Validity Index
ELS: Ethical Leadership Scale
IFI: Incremental Fit Index
ITC: International Test Commission
JS: Job Satisfaction
NFI: Normed Fit Index
PLBI: Perceived Leader Behavioral Integrity
RMSEA: Root Mean Square Error of Approximation
SRMR: Standardized Root Mean Square Residual
